# Ang-2 is a potential molecular marker for lymphatic metastasis and better response to bevacizumab therapy in ovarian cancer

**DOI:** 10.1007/s00432-023-05354-1

**Published:** 2023-09-08

**Authors:** Annabelle Volk, Karen Legler, Fabienne Hamester, Sascha Kuerti, Kathrin Eylmann, Maila Rossberg, Barbara Schmalfeldt, Leticia Oliveira-Ferrer

**Affiliations:** Department of Gynecology, University Medical Center Hamburg-Eppendorf, Martinistrasse 52, 20246 Hamburg, Germany

**Keywords:** Ovarian cancer, Ang-2, Bevacizumab, Lymphatic metastasis, Predictive marker

## Abstract

**Purpose:**

In ovarian cancer, there are two main routes of metastasis, namely intraperitoneal and retroperitoneal. Their biologic background is poorly understood. Identifying molecular markers involved might enable the development of tailored therapy regimens. Moreover, no reliable markers for response to anti-angiogenic treatment with bevacizumab are yet established. Angiopoietin-2 (Ang-2) is an angiogenic growth factor, involved in lymphatic activation and is associated with tumor progression. Here, we assessed the potential of Ang-2 as a molecular marker in metastasis and treatment of ovarian cancer.

**Methods:**

In our study, quantitative and qualitative protein Ang-2 expression in tumor tissue of ovarian cancer patients was analyzed by Western blot (*n* = 138) and immunohistochemistry (*n* = 58). Further, Ang-2 levels in blood samples were quantified in enzyme-linked immunosorbent assay (*n* = 38). Expression levels of different tumor spread patterns were evaluated, and survival analyses were made.

**Results:**

We observed that Ang-2 expression is significantly higher in tumors with retroperitoneal dissemination (pT1a–pT3b, pN1) compared to those showing intraperitoneal tumor growth (pT3c, pN0). In addition, patients with high Ang-2 expression have significantly longer overall survival compared to patients with low Ang-2 expression. Patients with high Ang-2 expression benefit significantly from therapy with bevacizumab.

**Conclusion:**

All in all, Ang-2 may serve as a molecular marker for patients with tumors prone to spread to lymph nodes and for patients who might benefit from bevacizumab therapy.

**Supplementary Information:**

The online version contains supplementary material available at 10.1007/s00432-023-05354-1.

## Introduction

Ovarian cancer is mostly diagnosed at an advanced tumor stage when tumor cells have already spread, resulting in a correspondingly poor prognosis for patients (Jelovac and Armstrong 2011). The most common routes of metastasis are intraperitoneal tumor growth and retroperitoneal lymphatic tumor spread (Rose et al. 1989). Hematogenous tumor dissemination is rarely observed (Yousefi et al. 2020; Pradeep et al. 2014). As there is growing evidence that different forms of metastasis have prognostic impact and are based on different biological backgrounds, elucidating the molecular mechanisms behind different tumor spread patterns becomes of greater importance (Prat 2015). The identification of specific molecular markers would allow applying individualized treatment regimens and might improve overall prognosis.

The publication of the LION trial in 2019 leads to challenge the previously commonly applied therapeutic principle of systematic pelvic and paraaortic lymphadenectomy, as it revealed that patients at a more advanced stage with macroscopically tumor free lymph nodes do not benefit from systematic lymphadenectomy (Harter et al. 2019). However, histopathological examination of lymph nodes is necessary in order to detect patients with retroperitoneal metastasis. These patients might benefit from a different treatment. Therefore, it is especially important to identify molecular markers that attribute the risk of lymphatic metastases.

Furthermore, maintenance therapy with PARP-inhibitors or antiangiogenic drugs, such as bevacizumab, is nowadays recommended in the national guideline for treatment of advanced ovarian cancer (Kuroki and Guntupalli 2020). Nonetheless, to this day there are no predictive biomarkers that help decide between different options of maintenance therapies (Colombo et al. 2019). Bevacizumab, the most commonly used antiangiogenic drug, has significant side effects, and not all patients respond to treatment (Burger et al. 2011; Haunschild and Tewari 2020). Thus, it is of great interest to enable identification of patients that benefit from bevacizumab therapy.

We could previously show that retroperitoneal tumor progression to lymph nodes is associated with high VEGF-C protein expression (Kuerti et al. 2017). Moreover, our research group recently revealed that VEGF-C serum levels might serve as a biomarker for evaluation of bevacizumab treatment response (Ding et al. 2022).

Similar to VEGF-C, angiopoietin-2 (Ang-2) is a widely known angiogenic growth factor. The angiopoietin family consists of Ang-1 to -4. These molecules are ligands to the tyrosine kinase receptors Tie1 and Tie2, forming one of the main pathways involved in angiogenesis. Ang-1 and -2 bind to Tie2, regulating angiogenesis and vascular remodeling with antagonistic effects (Akwii et al. 2019; Reiss 2010). While Ang-1 promotes stabilization of vessels, Ang-2 destabilizes vessels and promotes abnormal proliferation. Increased Ang-2 levels have been observed in different entities of human cancer (Nicolini et al. 2019). Interestingly, Ang-2 is also associated with the activation and remodeling of lymphatic vessels (Dellinger et al. 2008; Holopainen et al. 2012). Currently, trebananib, an anti-angiogenic drug that inhibits the binding of Ang-1 and -2 to Tie2, was tested in phase 3 clinical trials in ovarian cancer patients, resulting in prolonged progression-free survival (Monk et al. 2014). However, in combination with carboplatin and paclitaxel it was only minimally effective (Vergote et al. 2019).

In this study, we aimed to analyze the influence of tumoral Ang-2 level on lymph node involvement and retroperitoneal dissemination as well as its potential role as a biomarker to identify ovarian cancer patients who might benefit from bevacizumab maintenance therapy.

## Materials and Methods

### Patients

Samples from 205 patients with epithelial ovarian cancer treated at the University Medical Center Hamburg-Eppendorf were available. All patients underwent primary debulking surgery including lymphadenectomy between 1997 and 2019 and gave written informed consent to gain biomaterial and view their clinical records. This was approved by the local ethics committee “Ethik-Kommission der Ärztekammer Hamburg” (no. 04.05.2004). All experiments were performed in accordance with the relevant guidelines and regulations. Patient data were tracked from date of first diagnosis until October of 2020.

Patient cohorts for Western blot, immunohistochemistry (IHC) and enzyme-linked Immunosorbent assay (ELISA) analyses were classified with regard to tumor spread pattern as seen in Table 1.

**Table 1:** Patients characteristics

	Western blot		ELISA		IHC	
	*n* = 138	*n* (%)	*n* = 38	*n* (%)	*n* = 58	*n* (%)
Tumor spread pattern						
Retroperitoneal	pT1a–pT3b N1	25 (18.1)	pT1a–pT2c N1	15 (39.5)	pT1a–pT3b N1	10 (17.2)
Intraperitoneal	pT3c N0	31 (22.5)			pT3c N0	7 (12.1)
Mixed	pT3c N1	58 (42.0)			pT3c N1	32 (55.2)
Early stage	pT1a–pT3a N0	9 (6.5)	pT1a–pT2c N0	23 (60.5)	pT1a–pT3a N0	1 (1.7)
FIGO stage						
I		1 (0.7)		11 (28.9)		0
II		3 (2.2)		7 (18.4)		0
III		104 (75.4)		14 (36.8)		46 (79.3)
IV		24 (17.4)		2 (5.3)		7 (12.1)
Grading						
G1		6 (4.3)		8 (21.1)		3 (5.2)
G2		24 (17.4)		10 (26.3)		3 (5.2)
G3		105 (76.1)		20 (52.6)		49 (84.5)
pT						
pT1a		0		7 (18.4)		0
pT1b		0		3 (7.9)		0
pT1c		1 (0.7)		10 (26.3)		0
pT2				1 (2.6)		
pT2a		2 (1.4)		2 (5.3)		1 (1.7)
pT2b		2 (1.4)		8 (21.1)		1 (1.7)
pT2c		6 (4.3)		7 (18.4)		3 (5.2)
pT3a		6 (4.3)		0		1 (1.7)
pT3b		21 (15.2)		0		6 (10.3)
pT3c		98 (71.0)		0		44 (75.9)
pT4		0		0		0
Nodal involvement						
N0		42 (30.4)		23 (60.5)		9 (15.5)
N1		83 (60.1)		15 (39.5)		42 (72.4)
Histological type						
Serous/serous papillary		138 (100.0)		20 (52.6)		58 (100.0)
Others		0		18 (47.4)		0
Residual tumor after surgery						
No macroscopic tumor		95 (68.8)		35 (92.1)		37 (63.8)
< 1 cm		24 (17.4)		2 (5.3)		10 (17.2)
> 1 cm		16 (11.6)		0		11 (19.0)
Chemotherapy						
Platin-based		69 (50.0)		23 (60.5)		8 (13.8)
Platin-based + bevacizumab		69 (50.0)		12 (31.6)		49 (84.5)
No chemotherapy		0		3 (7.9)		1 (1.7)
Survival						
Alive		58 (42.0)		27 (71.1)		31 (53.4)
Deceased		80 (58.0)		8 (21.1)		27 (46.6)
Tumor recurrence						
Yes		110 (79.7)		10 (26.3)		49 (84.5)
No		28 (20.3)		23 (60.5)		9 (15.5)

### Tissue samples

All tissue samples were gained during surgery and were immediately stored in liquid nitrogen as fresh frozen samples, or fixed in 4% buffered formalin and subsequently embedded in paraffin as described before (Kuerti et al. 2017; Milde-Langosch et al. 2005).

### Protein extraction and Western blot

Only tissue samples consisting of at least 70% tumor cells, as quantified by H&E staining, were used for protein extraction. Protein extraction was performed as described previously (Trillsch et al. 2016).

For each sample 20 μg protein lysate was loaded per well. Equal amount of loading was verified by immunoblotting with β-actin antibody (SantaCruz, Dallas, Texas, USA, sc-47778, Lot E0919, 1:4000). Protein lysate from human pulmonary microvascular endothelial cells (HPMEC) served as positive control and reference sample. To separate the proteins, a 10% sodium dodecyl sulfate (SDS) gel was used. Electrophoresis was performed for approximately 18 h at 55 V. Next, the proteins were blotted for 2 h at 1.00 A to polyvinylidene difluoride membranes. Membranes were blocked with 5% non-fat dry milk for 1 h at room temperature in Tris-buffered saline (TBS) + 20% Tween (TBST) and subsequently incubated with a monoclonal Ang-2 antibody (SantaCruz, sc-74403, Lot B0916, 1:1000) overnight at 4 °C. Afterward, membranes were incubated with the corresponding secondary antibody (goat anti mouse, southern biotech, Birmingham, Alabama, USA, 1030-05, Lot K3515T566C, 1:8000) for 1 h at room temperature. In between incubation steps, membranes were washed with TBST. All antibodies were diluted in 1.5% non-fat dry milk in TBST. The detection was performed with chemiluminescence reagent (Westar Nova 2.0 CYANAGEN, Bologna, Italy, XLS071) to visualize the protein expression on FUJI-super RX medical X-ray films. Band intensities were quantified by densitometry (GS-700 Imaging Densitometer, Bio-Rad, Munich, Germany) and calculated as percentage of the HPMEC control sample.

### Immunohistochemistry

Paraffin sections of tumor samples were dehydrated in descending concentrations of ethanol and xylene. In between incubation steps, slides were washed twice with TBST and once with TBS. All antibodies were diluted in Antibody Diluent (Agilent, Carpinteria, California, USA, DAKO S0809).

For Ang-2 staining, slides were heated in antigen retrieval buffer (Agilent, DAKO S1699) in a pressure cooker for 10 min at 121 °C. Afterward, the tissue was blocked with 10% normal horse serum (Vector Laboratories, Newark, California, USA, S-2000) for 30 min at room temperature. The primary antibody for Ang-2 (SantaCruz, sc-74403, Lot A0813, 1:50) incubated over night at 4 °C. Peroxidase blocking was performed with 1% H_2_O_2_ in methanol for 30 min at room temperature.

For CD31 staining, the slides were pretreated in Target-Retrieval-Buffer pH9 (Agilent, DAKO S2367) for 20 min at 85 °C in a steamer. The primary antibody (Agilent, DAKO M0823, Lot 00054859, 1:60) was incubated over night at 4 °C. Peroxidase blocking was performed with 0.5% H_2_O_2_ in methanol for 30 min at room temperature.

Biotinylated secondary horse anti-mouse IgG (Vector Laboratories, BA-2000) was incubated 1 h at room temperature. For signal amplification, avidin–biotin complex (Vector Laboratories, PK-6100) was applied. A DAB staining kit (Vector Laboratories, SK-4100) was used, and slides were counterstained with hematoxylin. The tissue was rehydrated using ascending concentrations of ethanol and xylene. Placenta tissue served as positive control, while isotype control antibody (mouse IgG1, Agilent, DAKO X0931) served as negative control.

Slides were independently reviewed by two researchers. The Ang-2 staining intensity was scored on a scale from 0 (no staining), 1 (weak staining), 2 (moderate staining) to 3 (strong staining) and multiplied by the amount of tumor tissue that was stained from 1 (< 25%), 2 (25–50%), 3 (50–75%) to 4 (> 75%). Immunoreactive scores for Ang-2 tissue and vascular staining were created separately. For CD31 staining only the density of vessels was considered and assessed on a scale of 0 to 3.

### Serum samples and ELISA analysis

The patients’ blood was collected prior to therapy in serum-gel monovettes (Sarstedt, Germany) and was prepared as previously described (Ding et al. 2022). All serum samples were stored at − 80 °C and thawed on ice right before measurement. Serum Ang-2 was measured with the Human Angiopoietin-2 Quantikine ELISA set (R&D Systems, Minneapolis, Minnesota, USA, Catalog No. DANG-20). Serum samples were diluted 1:5. The procedure was performed according to protocol provided by the manufacturers.

### Statistical analyses

SPSS version 27.0 was used for statistical analyses. Different Ang-2 expression levels among cohorts were assessed by *t* test. Ang-2 expression was correlated by Pearson’s Chi-squared and Spearman test with clinical and pathological parameters. Survival curves were plotted using the Kaplan–Meier method, and differences between survival curves were tested using the log-rank test. Probability values less than 0.05 were regarded as statistically significant.

## Results

### Ang-2 expression analysis on tissue and serum samples of ovarian cancer patients

Initially, 175 patients were analyzed in Western blot. For statistical evaluation patients who received neoadjuvant or no chemotherapy at all (*n* = 15) as well as patients with a histology subtype other than serous/serous papillary (*n* = 22) were excluded from analyses. A total of 138 patients were left to be included.

Twenty-five (18.1%) patients in tumor stage pT1a–pT3b, pN1, suffering from lymph node metastases only and without significant intraperitoneal tumor growth, were included in the retroperitoneal group. In contrast, 31 (22.5%) patients in tumor stage pT3c, pN0, only suffering from intraperitoneal tumor growth without lymph node metastases, were assigned to the intraperitoneal group. The mixed group consists of 58 (42.0%) patients with both lymph node metastases as well as significant intraperitoneal tumor growth in tumor stage pT3c, pN1. A small group of 9 (6.5%) patients was diagnosed at an early tumor stage pT1a–pT3a, pN0.

**Fig. 1:**
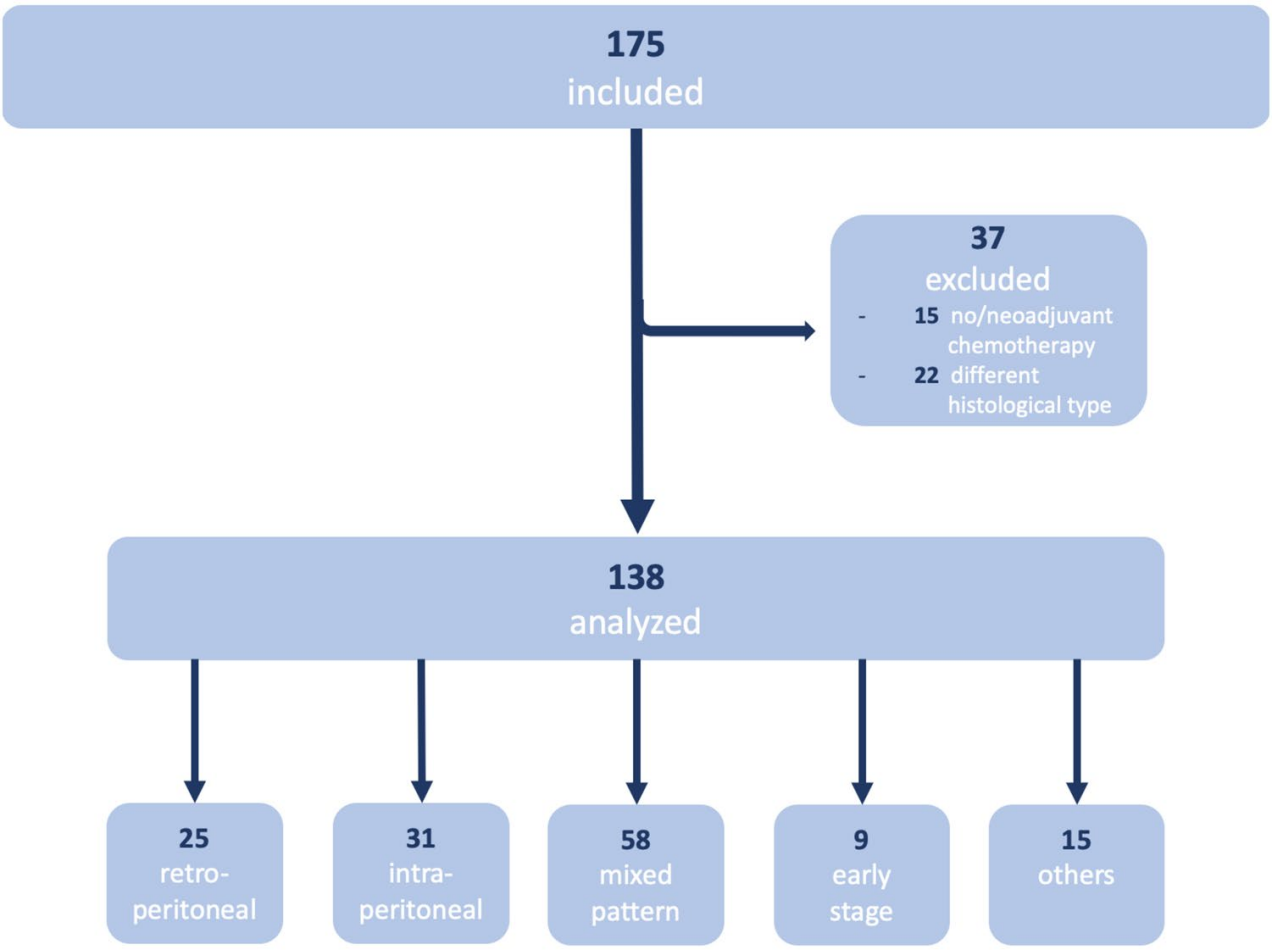
Consort flow diagram for Western blot analyses. 175 tumor samples were analyzed in Western blot. For statistical evaluation patients who received neoadjuvant or no chemotherapy at all (*n* = 15) as well as patients with a histology subtype other than serous/serous papillary (*n* = 22) were excluded from analyses. A total of 138 patients were left to be included and assigned to 4 groups according to their tumor spread pattern. 25 were assigned to the retroperitoneal group, 31 to the intraperitoneal group, 58 to the mixed group and 9 to the early-stage group. 15 patients had tumor spread patterns that could not be assigned to any of these four groups

Also, there were 15 patients with tumor spread patterns that could not be assigned to any of these four groups (Fig. 1).

**Fig. 2:**
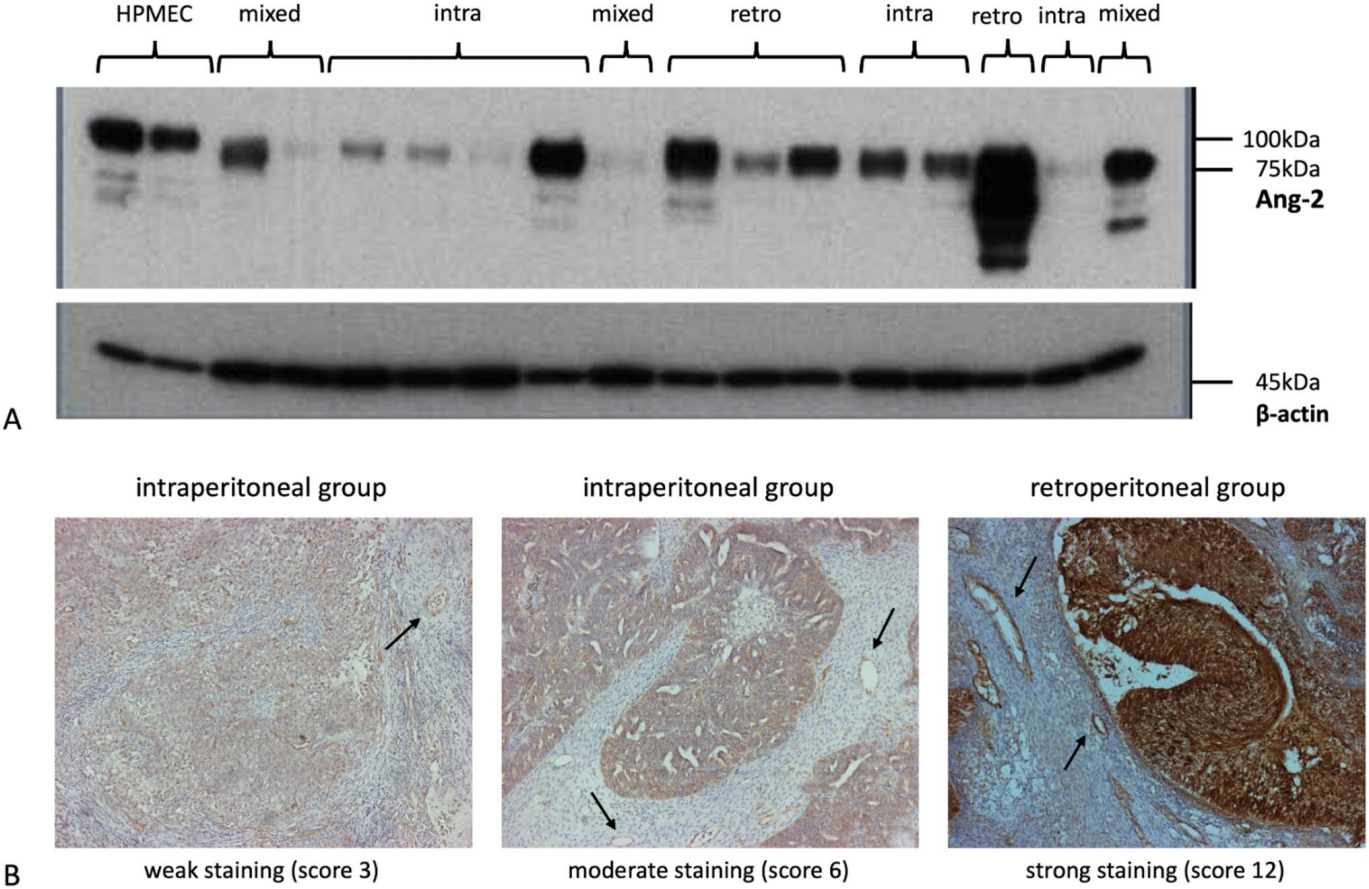
Ang-2 expression analysis on tissue samples of ovarian cancer patients. **A** In Western blot we detected an Ang-2 specific band at approximately 75 kDa. There was a heterogeneous Ang-2 expression within the patient cohort. The intensity of the signals, respectively the Ang-2 expression level, varied strongly depending on the metastasis pattern. **B** In IHC Ang-2 expression was found in the capillaries (marked by arrows) as well as in the tumor tissue itself. Again, a different degree of staining depending on the tumor metastasis pattern was noted

As seen in Fig. 2A, an Ang-2 specific band could be detected at approximately 75 kDa. The endothelial cell line HPMEC served as positive control. Corresponding bands of β-actin were detected at 45 kDa (Fig. 2A). There was a heterogeneous Ang-2 expression within the patient cohort. Defining the expression level of HPMEC at 100%, the expression level of analyzed tumors ranged from 7 to 15,962%. Two patients did not express Ang-2. The median expression level of the overall cohort was 1876%.

As protein lysates used in Western blot analyses did not solely contain carcinoma cells and as Ang-2 is also expressed by vascular endothelium, we performed IHC with paraffin sections of 58 tumors to visualize the distribution and Ang-2 expression intensity in the different tumor tissue compartments. Four slides were not analyzable because of insufficient quality and were therefore excluded from the analysis. Representative images of variously strong stained tumors are shown in Fig. 2B. Ang-2 expression was detected at different intensities in tumor cells, as well as in endothelial cells of tumor associated vessels (arrows, Fig. 2B). IHC scores ranged from 0 to 12. Weak staining was defined from 1 to 4, moderate staining from 5 to 8 and strong staining from 9 to 12. Regarding the staining of tumor tissue, 20 (37.04%) tumors were stained weakly, 22 (40.74%) moderately and 9 (16.67%) strongly. Regarding vascular staining, 20 (37.04%) slides were stained weakly, 11 (20.37%) moderately and 18 (33.33%) strongly.

Additionally, CD31 staining was performed to assess the vascular density on the corresponding tumors. Scores for CD31 ranged from 1 to 3, with 4 (7.02%) tumors with low vessel density, 38 (66.67%) with intermediate and 15 (26.32%) with high density.

Ang-2 staining of tumor cells did not correlate with vascular density, determined by CD31 staining (*p* = 0.986). Vascular density and endothelial Ang-2 expression did not correlate either (*p* = 0.461). However, Ang-2 tumor cell staining correlated with Ang-2 vascular staining (*p* < 0.001). Interestingly, when comparing Western blot and IHC expression levels, Ang-2 levels in protein lysates only correlated with Ang-2 vascular staining (*p* = 0.001), whereas no correlation between Ang-2 tissue staining and Western blot expression levels was noted (*p* = 0.625).

Serum samples of 38 patients with ovarian cancer at tumor stage pT1a-pT2c were analyzed. A total of 15 patients (39.5%) whose tumors had already spread to lymph nodes (pT1a–pT2c, pN1) were assigned to the retroperitoneal group, while 23 patients (60.5%) were diagnosed at an early stage without lymph node metastases (pT1a–pT2c, pN0) (Table 1). Serum Ang-2 levels ranged from 860.3 to 6114, with a median expression level of 1902.5. Later on, one patient from the early stage group with unusual high levels of serum Ang-2 was excluded. Clinical records revealed that the patient concerned suffered from ulcerative colitis. In 2006 Koutroubakis et al. revealed that Ang-2 serum levels are elevated in patients with inflammatory bowel disease, leading us to exclude said patient (Koutroubakis et al. 2006).

### Ovarian cancer tissue with retroperitoneal spread expresses significant higher Ang-2 levels

Ang-2 expression in Western blot varied strongly depending on the tumor spread pattern observed in the patient. Defining high Ang-2 levels above the median, 20 (80%) patients of the retroperitoneal group expressed high levels. In comparison, 16 (51.61%) of the intraperitoneal group, 27 (46.55%) of the mixed group and 3 (33.33%) of the early stage group expressed high Ang-2 levels.

**Fig. 3:**
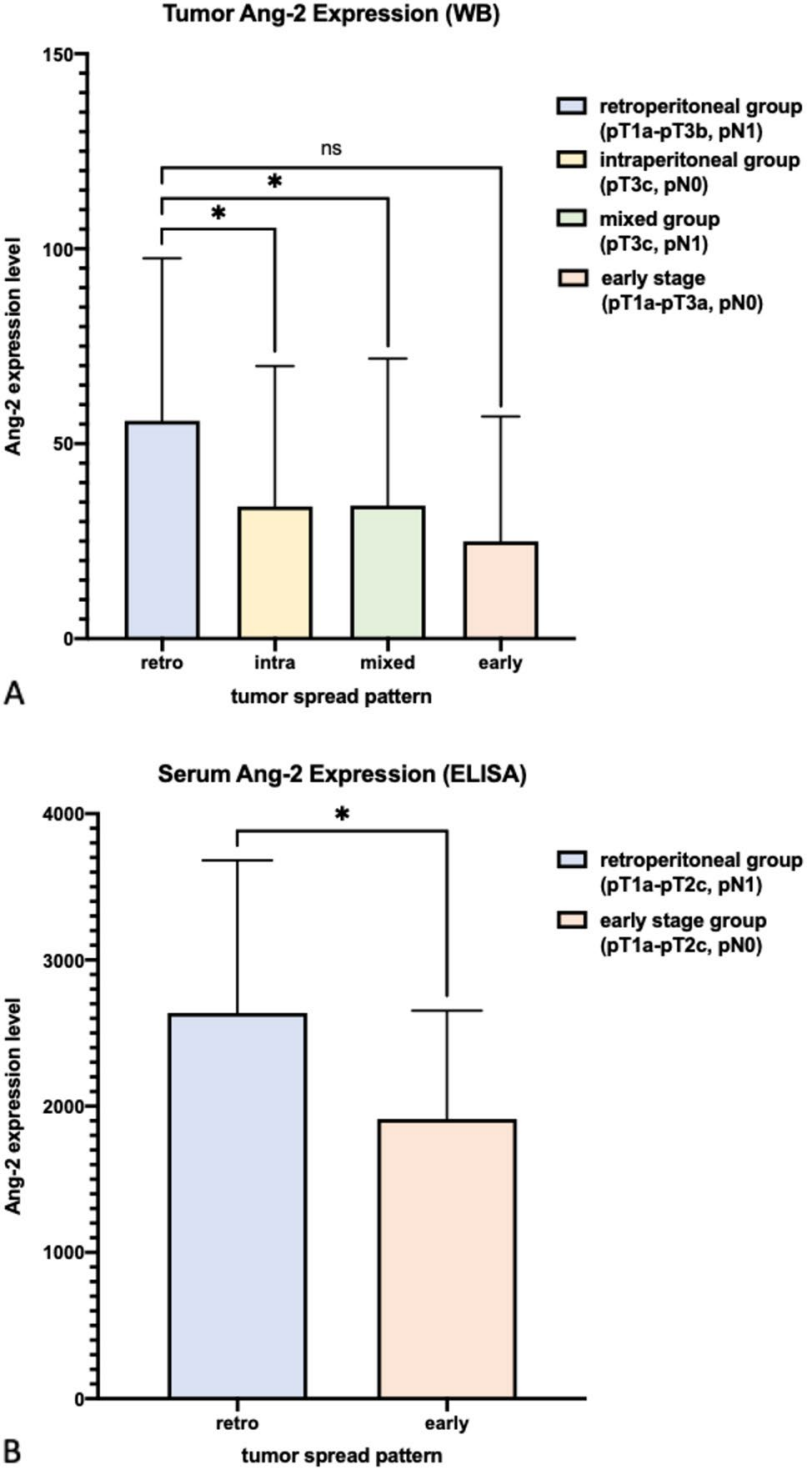
Ang-2 expression analysis in tumor tissue/serum according to tumor spread pattern. **A** Ang-2 expression is significantly higher in tumors with retroperitoneal metastasis (pT1a–pT3b, pN1) compared to tumors with intraperitoneal metastasis only (pT3c, pN0; *p* = 0.039) and compared to tumors with a mixed metastatic pattern (pT3c, pN1; *p* = 0.022). Tumor tissue of the retroperitoneal group expresses higher Ang-2 levels compared to tumor tissue of the early group, without reaching statistical significance (*p* = 0.053). **B** In serum of ovarian cancer patients with early stage tumors and without intraperitoneal metastasis (pT1a–pT2c), Ang-2 expression is significantly higher in tumors with lymph node metastases (pN1) compared to tumors without (pN0; *p* = 0.029)

The statistical analyses revealed a significantly higher Ang-2 expression in the retroperitoneal group compared to the intraperitoneal group (mean 55.85 vs. 33.91, *p* = 0.039, Fig. 3A). Also, Ang-2 expression in tumors with a retroperitoneal spread pattern was significantly higher compared to tumors with mixed type of metastasis (mean 55.85 vs. 34.10, *p* = 0.022, Fig. 3A). A similar trend could be detected between the retroperitoneal group and tumors diagnosed at an early stage. Mean expression levels of these tumors were lower compared to those of the retroperitoneal group (mean 55.85 vs. 24.94). However, these findings did not reach statistical significance (*p* = 0.053, Fig. 3A).

Between all the other groups, no significant differences in expression levels were found.

In line with these data, a significantly higher Ang-2 expression in serum samples was noted in the retroperitoneal group compared to the tumors diagnosed at an early stage without lymph node metastases (mean 2638 vs. 1913, *p* = 0.029, Fig. 3B).

### Ang-2 expression correlates significantly with postoperative residual tumor

Patients were divided into two groups according to the median Ang-2 expression level, defining high (> median) and low (< median) levels. The postoperative residual tumor, which was categorized into “no postoperative tumor residual”, “tumor residual < 1 cm” and “tumor residual > 1 cm”, correlates significantly with Ang-2 expression in the Western blot cohort. Here, lower Ang-2 levels were detected in patients with more residual tumor after surgery (*p* = 0.022, Supplementary Fig. 1A). Further analyses revealed no significant differences of Ang-2 expression levels in tumor stage (pT, pM) nor histological grading (Supplementary Fig. 1B–E). However, dividing the patient cohort according to quartiles, we observed high Ang-2 expression levels (= quartile 4) in tumors with positive lymph nodes (*p* = 0.060, Supplementary Fig. 1E). As most of our patients were diagnosed at an advanced tumor stage (FIGO IIIC-IV; *n* = 116) and due to a small number of patients diagnosed at an early stage (FIGO I-IIA; *n* = 2), no correlation analyses were performed in regard to FIGO stage.

In IHC as well as in ELISA there were no significant correlations between Ang-2 level and clinical or pathological characteristics. No correlation with CA-125 expression levels could be detected (data not shown).

### Patients with high Ang-2 expression had a significantly longer overall survival

For survival analyses, the overall Western blot patient cohort was divided into two same sized groups according to the median Ang-2 expression value. The median follow-up of the overall cohort (*n* = 138) was 43 months.

**Fig. 4:**
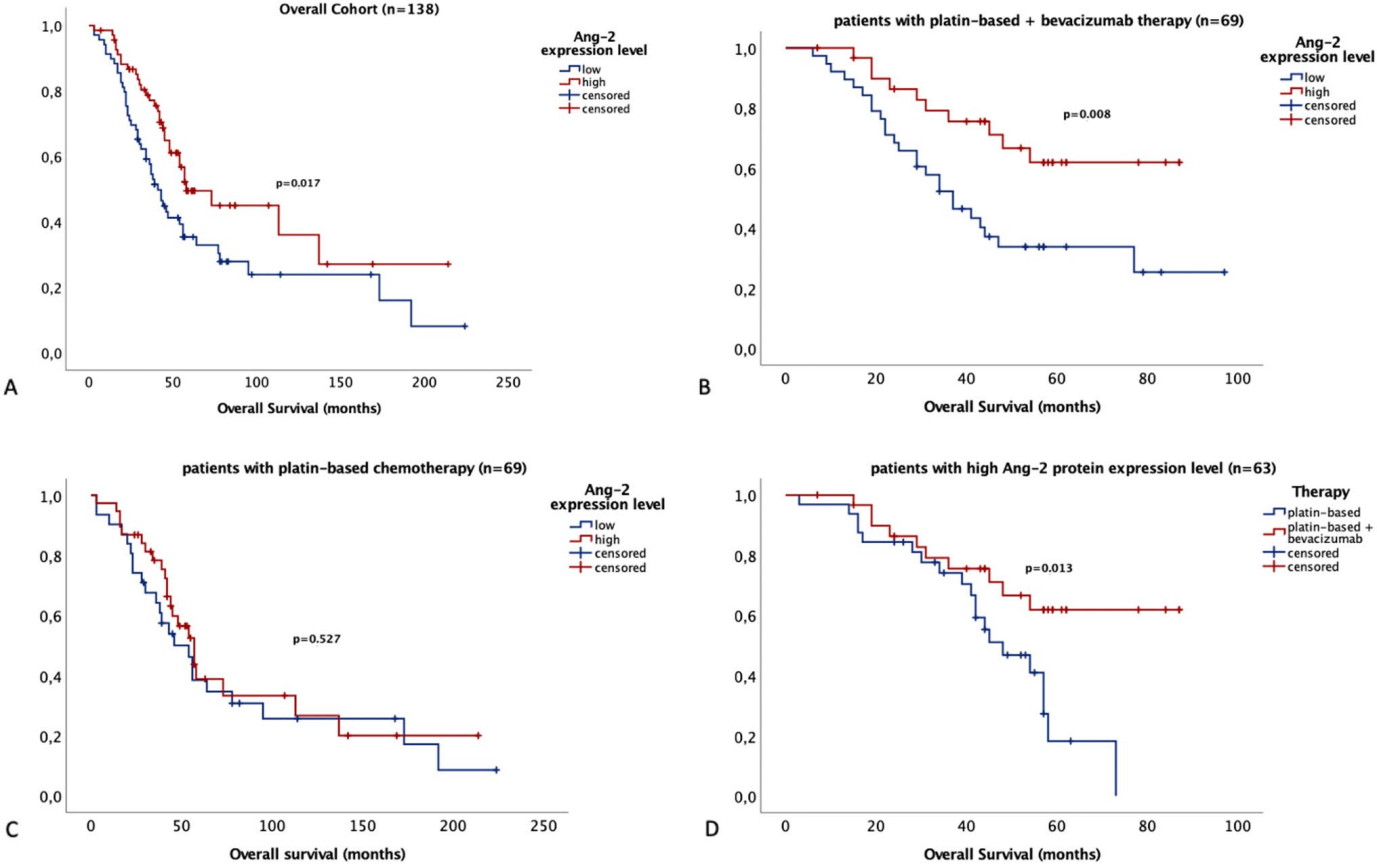
Overall survival according to Ang-2 expression in Western blot and treatment with or without bevacizumab. **A** In the overall cohort high Ang-2 expression correlates significantly with longer overall survival (*p* = 0.017). **B** After dividing the cohort with respect to therapy regimens, a significant association is only seen in patients treated with bevacizumab therapy (*p* = 0.008). **C** In patients that did not receive bevacizumab therapy, Ang-2 expression levels have no significant impact on overall survival (*p* = 0.527). **D** Moreover, in the cohort of ovarian cancer patients with high Ang-2 expression, patients treated with bevacizumab show significantly longer overall survival (*p* = 0.013)

No significant association between Ang-2 expression and progression free survival (PFS; *p* = 0.134, Supplementary Fig. 2A–C) was found, whereas patients with high Ang-2 expression levels showed significantly longer overall survival rates (OAS; *p* = 0.017, Fig. 4A). Interestingly, stratified survival analyses revealed that the prognostic effect of Ang-2 was restricted to the subgroup of patients treated with bevacizumab as maintenance therapy.

Here, high Ang-2 levels correlated significantly with longer OAS (*p* = 0.008, Fig. 4B), while no prognostic difference was observed in the cohort of patients receiving platinum-based therapy alone with respect to Ang-2 expression levels (*p* = 0.527, Fig. 4C).

Indeed, patients showing high Ang-2 expression levels did benefit from antiangiogenic therapy. As shown in Fig. 4D, patients treated with platin-based therapy alone had significantly shorter OAS than those who subsequently received bevacizumab maintenance therapy (*p* = 0.013, Fig. 4D). In contrast, no significant difference in OAS was observed between the two treatment arms in patients with low Ang-2 expression levels (*p* = 0.737; data not shown). Similar trends were found in PFS analyses, where high Ang-2 expression levels are associated with longer PFS in the cohort of patients treated with bevacizumab. However, this correlation did not reach significance (*p* = 0.069, Supplementary Fig. 2C).

In multivariate Cox regression analysis, including Ang-2 expression levels and clinical prognostic parameters as FIGO stage and nodal status, postoperative residual tumor is the only independent and significant prognostic indicator (*p* = 0.001). Ang-2 expression did not turn out to be an independent prognostic indicator (*p* = 0.157, data not shown).

**Fig. 5:**
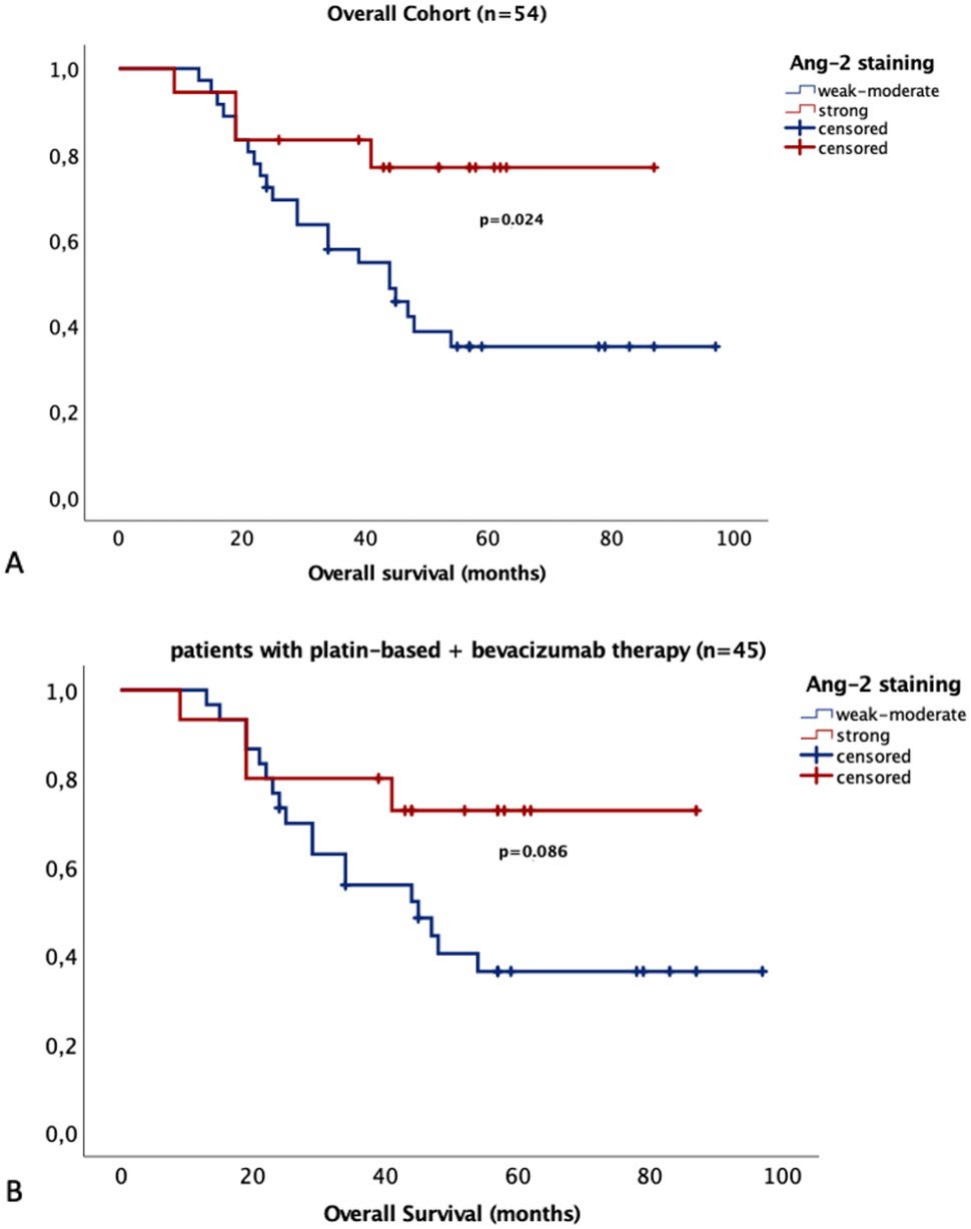
Overall survival according to Ang-2 expression in IHC and treatment with or without bevacizumab. **A** In the overall cohort, high Ang-2 expression in tumor capillaries correlates significantly with longer overall survival (*p* = 0.024). **B** In the cohort of patients with bevacizumab therapy, patients with high Ang-2 expression in the tumor capillaries have a longer overall survival without reaching statistical significance (*p* = 0.086)

In addition, survival analyses in the IHC cohort were performed. As stated before, only vascular staining scores in IHC correlated with Western blot expression levels. As vascular Ang-2 expression was therefore assumed to be responsible for the biggest share of expression levels, only scores of Ang-2 vascular staining were used for analyses. Patients were divided into two groups. Tumors with weak to moderate staining (score 1–8; *n* = 36; 66.7%) were compared to tumors with strong staining (score > 8; *n* = 18; 33.3%). In line with the Western blot results, strong Ang-2 staining correlated with significantly longer OAS (*p* = 0.024, Fig. 5A). A similar trend could be shown in patients treated with platin-based-chemotherapy and bevacizumab (*p* = 0.086, Fig. 5B). Again, no statistical significance was reached in regard to Ang-2 staining in PFS (*p* = 0.336, Supplementary Fig. 3A).

In ELISA analyses, Ang-2 serum levels did not correlate with OAS or PFS (Supplementary Fig. 3B, C).

## Discussion

Ovarian cancer spreads mainly intraperitoneally and/or retroperitoneally. Although we assume that these two forms of dissemination are based on different biological backgrounds and could potentially be treated in a differentiated manner, the molecular mechanisms behind these two subtypes have been little explored.

In this study, we have identified Ang-2 as a potential marker for retroperitoneal tumor growth. Using Western blot analyses, significantly higher Ang-2 expression levels were found in tumors with retroperitoneal metastasis (pT1a–3b, pN1) compared to those showing intraperitoneal tumor growth only (pT3c, pN0). Interestingly, tumors with both intraperitoneal tumor growth and lymphatic metastases (mixed cohort), at a more advanced tumor stage (pT3c, pN1), expressed significantly lower levels of Ang-2. This might indicate that Ang-2 could serve as a marker for early retroperitoneal tumor spread in ovarian cancer.

Indeed, using a commercial ELISA, we could detect significantly elevated serum Ang-2 levels in ovarian cancer patients at an early tumor stage (pT1a–pT2c) with lymphatic metastases. This has also been shown in other entities of cancer, for example, gastric and lung cancer, where Ang-2 levels are associated with lymph node involvement (Jo et al. 2009; Xu et al. 2017).

Even though measuring Ang-2 serum levels would be a practicable non-invasive method to assess the risk of lymph node metastases in early ovarian cancer, it has to be taken into account that Ang-2 serum expression levels are also affected by other pathologies. For example, widely spread cardiovascular diseases have a strong impact on Ang-2 serum levels (Nicolini et al. 2019). In 2005, Nadar et al. suggested that patients suffering from arterial hypertension expressed comparably higher Ang-2 levels than normotensive patients, making Ang-2 serum levels slightly vulnerable (Nadar et al. 2005).

Our research group has recently shown that high VEGF-C expression and simultaneously low E-Cadherin expression can be found in tumors with retroperitoneal tumor growth. More precisely, we detected high VEGF-C levels in patients with solely retroperitoneal spread while tumors of patients with intraperitoneal tumor growth, regardless of lymph node status, expressed lower VEGF-C levels (Kuerti et al. 2017). Remarkably, the identical observation has been made for Ang-2 in the present study. Taking these observations into account, we strongly believe that tumors with retroperitoneal metastasis represent a distinct biological subgroup that might benefit from different therapy regimens. In our opinion, having a reliable marker for early retroperitoneal tumor spread might help tailor therapeutic strategies.

Furthermore, a significantly longer OAS for patients with high tumoral Ang-2 expression was observed, especially in the subgroup that received bevacizumab as maintenance therapy. In fact, patients with high tumoral Ang-2 levels have a significantly better prognosis when treated with bevacizumab, while patients with low tumoral Ang-2 levels did not benefit from the anti-angiogenic therapy. This suggests that Ang-2 might serve as a predictive marker for identification of patients that could benefit from maintenance therapy with bevacizumab.

As Western blot is not a commonly used clinical diagnostic tool, the IHC data are of greater clinical interest. Here, we could corroborate the predictive value of Ang-2 and note a strong trend toward longer OAS in patients with high Ang-2 levels treated with bevacizumab. Still, this association did not reach significant levels, which might be due to the small size of the IHC cohort.

Our findings on the predictive value of Ang-2 could not be confirmed in our ELISA cohort, in which an even smaller number of samples was available. Actually, Alvarez Secord et al. published a study in 2020 including 751 patients, showing no predictive value of Ang-2 expression in blood samples for PFS nor OAS for patients treated with bevacizumab (Alvarez Secord et al. 2020). In conjunction with our findings, this suggests that the predictive role of Ang-2 is restricted to its expression in the tumor compartment, implying that Ang-2 might serve as a tissue-based biomarker but is not suitable as a blood-based biomarker. We assume that the Ang-2 levels secreted by endothelial cells from tumor-associated blood vessels are sufficient to efficiently modulate the angiogenic balance in the tumor environment and influence the effect of bevacizumab, but that Ang-2 may not be released into the circulation to the same extent and therefore may not reflect the conditions in the tumor compartment. Therefore, analyses of Ang-2 expression on tumor tissue using IHC and including larger cohorts might be of great interest to validate the predictive role of Ang-2 in ovarian cancer patients treated with bevacizumab therapy.

Our findings in PFS analyses did not reach significance. Nevertheless, in our retrospective study design, the detection of disease progression depends on how early patients consult their doctors and is therefore determined by symptom burden. As this is no prospective study design where patients are observed under a strict protocol with, For example, CT scans, OAS analyses are a more reliable parameter than PFS analyses.

In our IHC analyses, the vascular density assessed by CD31 staining did not correlate with Ang-2 staining of tumor tissue, leading to the assumption that vascular density in ovarian cancer tissue is not primarily dependent on Ang-2 expression. In accordance with these findings, Hashizume et al. did not note changes in tumor vascularity in mouse-model experiments of selective Ang-2 blocking. However, after VEGF blocking, vascular density tended to be less and was strongly reduced after combination of both (Hashizume et al. 2010). This suggests that other angiogenic factors, such as VEGF, might have a stronger impact on vascular density in cancer tissue.

Interestingly, the Ang-2 expression in tumor cells correlated with the vascular Ang-2 expression, which suggests that high tumoral Ang-2 expression leads to angiogenic activation of surrounding blood vessels. As only Ang-2 vascular staining correlated with Western blot Ang-2 expression levels, we assume that the biggest share of Ang-2 is expressed by endothelial cells of tumor vessels. In line with this assumption, other studies have shown that Ang-2 expression was mainly limited to the endothelial cell compartment in cancer tissue (Goede et al. 2010).

Additionally, we found a significant association between postoperative residual tumor and Ang-2 expression in the Western blot cohort, with lower Ang-2 levels in tumors that could not be removed completely during surgery. As lower Ang-2 levels were predominantly found in tumors that had spread throughout the peritoneal cavity and higher levels correlated with tumor growth restricted to the retroperitoneal lymph nodes, this might be related to intraperitoneal metastases being a greater challenge to be removed completely (Horowitz et al. 2015; Coccolini et al. 2013). However, these findings could not be corroborated in the IHC or ELISA data.

Taken together, the identification of molecular markers for tumor spread patterns and response to anti-angiogenic therapies are required to customize therapy regimens and therefore improve prognosis in ovarian cancer patients. Ang-2 is a candidate marker for retroperitoneal lymphatic tumor spread and for better outcome of ovarian cancer patients treated with bevacizumab.

A limitation of this study is the heterogeneous therapy regimens applied in our overall cohort. Patients were included over a long period of time, from 1997 to 2020, and bevacizumab therapy was only applied after 2011 (Burger et al. 2011). Besides, patients who suffer from severe secondary diseases or have other contra indications are not considered for anti-angiogenic therapy. In this context, patients enrolled in the bevacizumab cohort had a higher life expectancy overall. Also, as ovarian cancer is usually diagnosed at an advanced stage, naturally the cohort of patients with early stage ovarian cancer was rather small.

Moreover, these promising results are based on quantitative and qualitative expression analyses of retrospectively analyzed tumor samples, making further prospective analyses necessary.

### Supplementary Information

Below is the link to the electronic supplementary material.

Supplementary file1 (DOCX 10661 kb)

## Data Availability

The dataset generated and analyzed during the current study are available from the corresponding author on reasonable request.
