# Supplementary material for: Ang-2 is a potential molecular marker for lymphatic metastasis and better response to bevacizumab therapy in ovarian cancer
Source: J Cancer Res Clin Oncol. 2023 Sep 8;149(17):15957–67. doi: 10.1007/s00432-023-05354-1 (PMC10620258; doi:10.1007/s00432-023-05354-1)
Supplement: Supplementary file 1 — Supplementary file1 (DOCX 10661 kb) [file 432_2023_5354_MOESM1_ESM.docx]

**A B**

**C D**

**
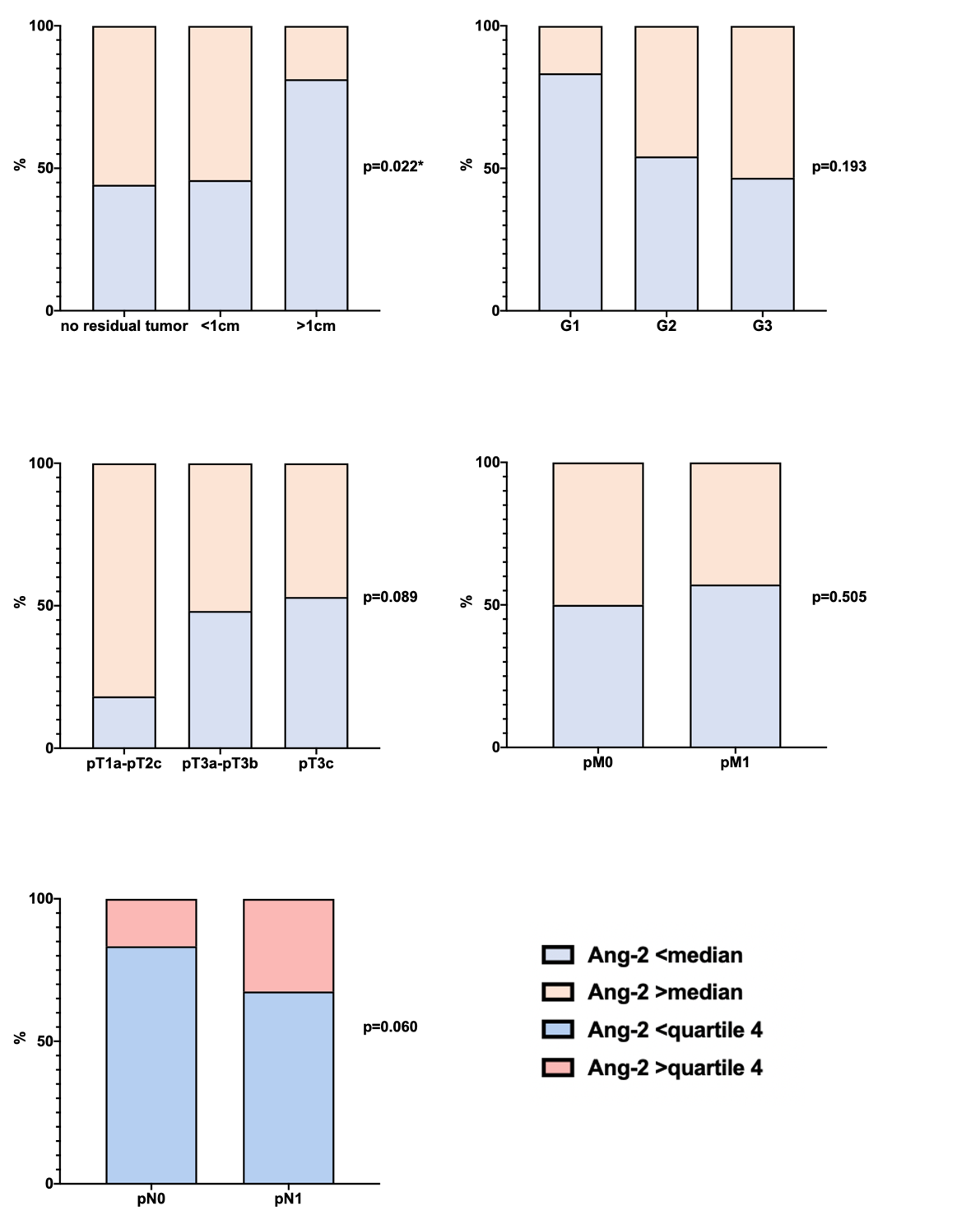

E**

**Supplementary Figure 1: Correlation between clinical or pathological parameters and Ang-2 expression.** (A) Postoperative residual tumor, which was categorized in „postoperative no tumor residual“, „tumor residual <1cm“ and „tumor residual >1cm“, correlates with Ang-2 expression, with lower Ang-2 levels in patients with more residual tumor after surgery (p=0.022). Analyses revealed no significant differences in Ang-2 expression levels in (B) histological grading (p=0.193), (C) tumor stage (p=0.089), (D) distant metastases (p=0.505) or (E) lymph node status (p=0.060).

**A**
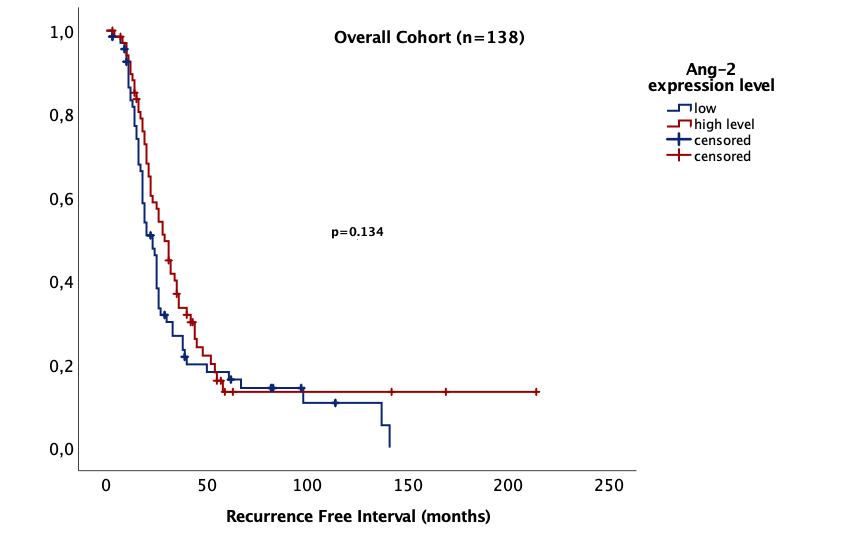


**B**
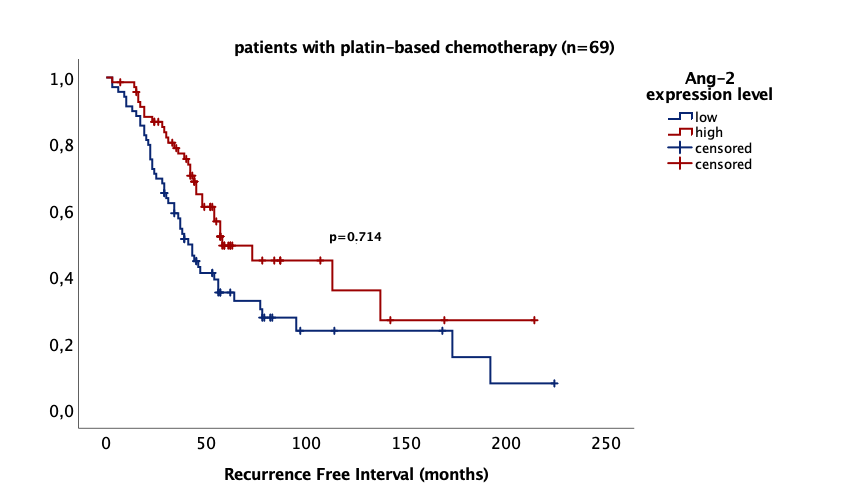


**C**
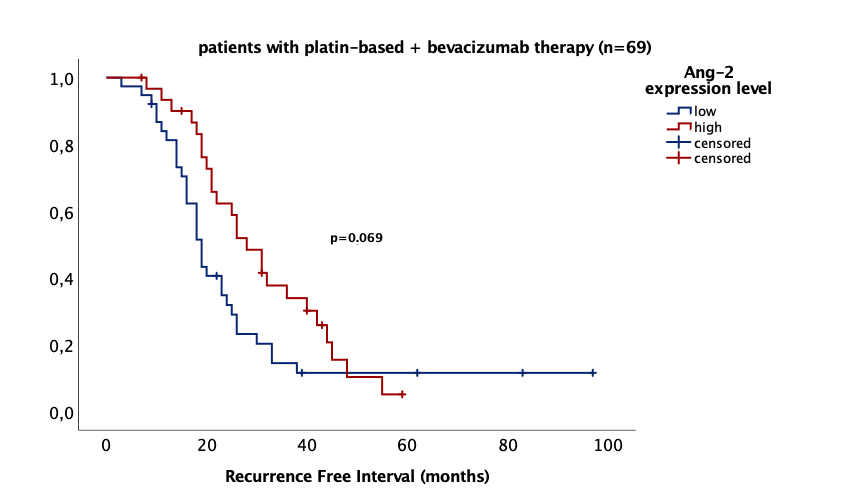


**Supplementary Figure 2: Progression free survival according to Ang-2 expression in Western Blot and treatment with or without bevacizumab.** (A) In the overall cohort Ang-2 expression does not correlate with longer progression free survival (p=0.134). (B) After stratifying the cohort with respect to therapy regimens, Ang-2 expression levels have no significant impact on progression free survival in patients that did not receive bevacizumab therapy (p=0.714), (C) or those that were treated with bevacizumab therapy (p=0.527).

**
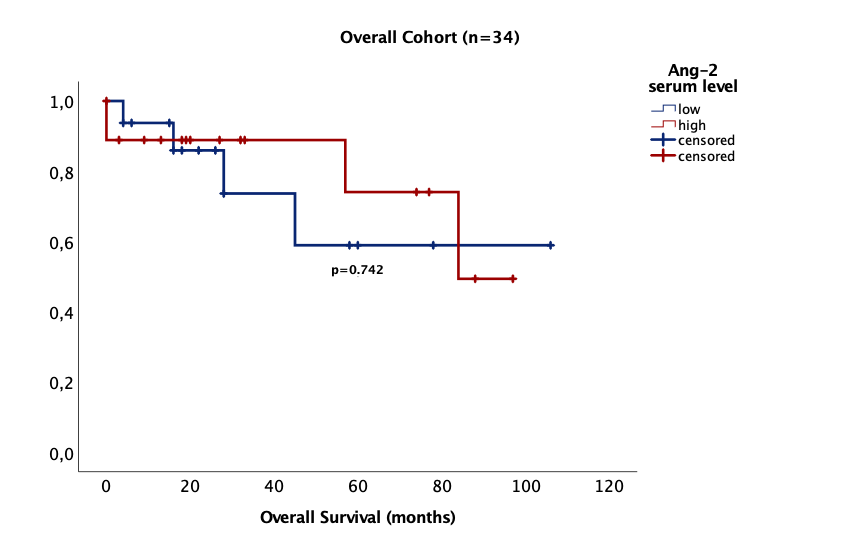
A
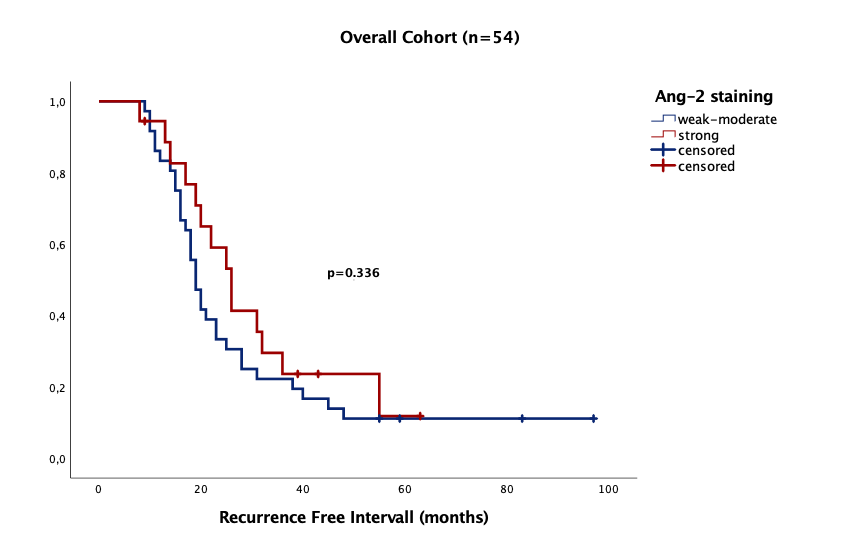
**

**B**

**
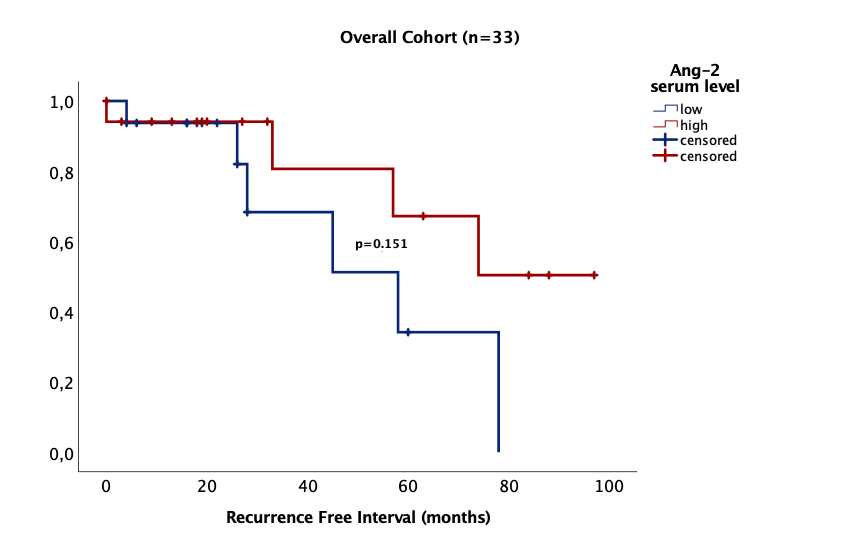
**

**C**

**Supplementary Figure 3: Overall and progression free survival according to Ang-2 expression in IHC and ELISA.** (A) Ang-2 expression in tumor capillaries does not correlate with longer progression free survival (p=0.336). (B) Ang-2 expression in serum samples does not correlate with longer overall survival (p=0.742), (C) or progression free survival (p=0.151).
